# Precision in Practice: Enhancing MI-E Therapy Evaluation for Improved Patient Outcomes, Excluding Speech and Swallowing. Reply to Allen et al. Comment on “Chatwin et al. Waves of Precision: A Practical Guide for Reviewing New Tools to Evaluate Mechanical In-Exsufflation Efficacy in Neuromuscular Disorders. *J. Clin. Med.* 2024, *13*, 2643”

**DOI:** 10.3390/jcm13174992

**Published:** 2024-08-23

**Authors:** Michelle Chatwin, Jesus Sancho, Manel Lujan, Tiina Andersen, Joao-Carlos Winck

**Affiliations:** 1 Neuromuscular Complex Care Centre, The National Hospital for Neurology and Neurosurgery, University College London Hospitals Foundation Trust, London WC1N 3BG, UK; michellechatwin@ymail.com; 2 Clinical and Academic Department of Sleep and Breathing, Royal Brompton Hospital, Part of Guys and St Thomas’ NHS Foundation Trust, London SW3 6NP, UK; 3 Respiratory Medicine Department, Hospital Clínico Universitario, 46010 Valencia, Spain; jesus.sancho@uv.es; 4 Institute of Health Research INCLIVA, 46010 Valencia, Spain; 5 Servei de Pneumologia, Parc Taulí Hospital Universitari, Institut d’Investigació i Innovació Parc Taulí (I3PT-CERCA), Universitat Autònoma de Barcelona, 08208 Sabadell, Spain; mlujan@tauli.cat; 6 Centro de investigación Biomédica en Red (CIBERES), 28029 Madrid, Spain; 7 Norwegian Advisory Unit on Home Mechanical Ventilation, Thoracic Department, Haukeland University Hospital, 5021 Bergen, Norway; tiina.maarit.andersen@helse-bergen.no; 8 The Faculty of Health and Social Sciences, Western Norway University of Applied Sciences, 5063 Bergen, Norway; 9 Cardiovascular R&D Centre (UniC), Faculdade de Medicina da Universidade do Porto, 4200-319 Porto, Portugal; 10 Pulmonology Unit, Instituto CUF, 4460-188 Porto, Portugal

We would like to thank Allen et al., [1] for their in-depth review of our manuscript ‘Waves of Precision: A Practical Guide for Reviewing New Tools to Evaluate Mechanical In-Exsufflation Efficacy in Neuromuscular Disorders’ [2]. We are pleased that the manuscript has fostered engagement and discussion. This work was created by three medical doctors and two physiotherapists who are experts in their fields, utilizing these tools in their daily practice, and describes how the medical and respiratory (physio) therapy profession is adopting new tools to refine the efficacy of mechanical insufflation-exsufflation (MI-E) treatments. This manuscript serves as a collective review, wherever possible, based on the available evidence for tools to assess cough efficacy with MI-E. 

Speech and language therapists Allen and Clunie [1] emphasize the role of the upper airway in swallowing and speaking during the assessments described in our paper. It is important to note that the upper airway serves multiple functions beyond speech and swallowing. While the primary function of the larynx in primitive species is to protect the lungs for aspiration with an adduction, the development of the human larynx has also led to a mechanism that facilitates well ventilation with an abduction. Further, a demand for phonation required the larynx to be able to perform fine-tuned rapid movements of adduction and abduction [3,4]. Consequently, the upper airway intersects with the upper gastrointestinal tract, requiring a central laryngeal position that balances its protective functions with ventilation, phonation, and swallowing. It is important to distinguish which functions are being evaluated. We believe that combining the evaluation of swallowing and phonation with airflow detection during MI-E therapy is a step in the wrong direction.

Allen et al., acknowledge that cervical auscultation can indicate changes in airflow [1]. We highlight the fact that you can listen to the sounds of breathing (i.e., changes in airflow) and the closing of the vocal cords during a cough by placing a stethoscope on the side of the neck near the larynx. Listening to these sounds can identify changes in the airflow during MI-E such as whether airflow is present, absent, reduced, or if there is an explosive expiratory flow during the cough, indicating the natural cough mechanism [5]. We would like to reiterate that we are not using cervical auscultation to assess swallowing during MI-E. When listening to airflow, either with or without MI-E, this is carried out throughout the treatment rather than just for one cycle, providing a more accurate representation of what is happening during treatment and minimizing any ‘artifacts’ such as swallowing, breathing pattern changes, or loss of contact of the stethoscope. Medical doctors and respiratory physiotherapists are highly qualified in listening to airflow, and, in our opinion and experience, can differentiate between the artifacts that Allen et al., describe [1]. Cervical auscultation is one tool among several in our paper that can help assess the effectiveness of MI-E and can be used in conjunction with other tools if needed [2].

As the authors comment, in the absence of empirical evidence, expert opinion plays an important role. In this way, we have a wide range of experience in the evaluation and management of respiratory problems in neuromuscular disorders, mainly in those with bulbar dysfunction including the use of ultrasound (US) to assess upper airway responses to non-invasive ventilation and MI-E [6,7]. We appreciate the author’s recommendation to visualize the vocal cords using the thyrohyoid and cricothyroid membrane as a window to avoid interference from calcifications of thyroid cartilage. We would like to make a few considerations in this regard. The calcification of the thyroid cartilage is indeed common (observed in up to 50% of cases), with no difference between genders [8]; however, this fact does not render the use of US infeasible [9]. It is important to note that most authors, including those cited by the authors of the letter to the Editor, [10,11,12,13] recommend access through thyroid cartilage to visualize the vocal cords, with the thyrohyoid membrane being more suitable for identifying the epiglottis [14]. Moreover, the studies referenced by the authors [10,14,15] do not evaluate thyroid calcifications. In this sense, we cordially invite the authors to conduct research assessing the effectiveness of the different access windows for visualizing the vocal cords in persons with neuromuscular disorders.

We are also grateful for the considerations made about the figures in our article, bearing in mind that the images were supervised by an expert radiologist. While the quality of the figures can indeed be improved, it is important to note that true vocal cords are hypoechoic, while false vocal cords are more hyperechoic and are positioned more caudally in relation to true vocal cords, as reflected by various authors, [10,13,16,17] contrary to your approach. Additionally, the legends of the figures in our article refer to movement, rather than obstruction. MI-E can produce total or partial obstruction in the upper airway, which can also modify the peak cough flow generated [18,19]. Here, we provide additional figures showing the upper airway responses to MI-E in persons with neuromuscular disorders with varying degrees of bulbar dysfunction (Figure 1, Figure 2 and Figure 3).

In conclusion, we thank Allen et al., for highlighting their clinical practice, which differs from ours as medical doctors and respiratory (physio) therapists. We highlight that cervical auscultation is used during many breath cycles to determine if airflow is present or absent and not to assess speech and swallowing. Until there is an evidence base to suggest their method of cervical ultrasound, for which we highlight the limitations in this letter, is superior, we believe our recommendations for the assessment of the efficacy of MI-E with ultrasound and auscultation are valid.

## Figures and Tables

**Figure 1:**
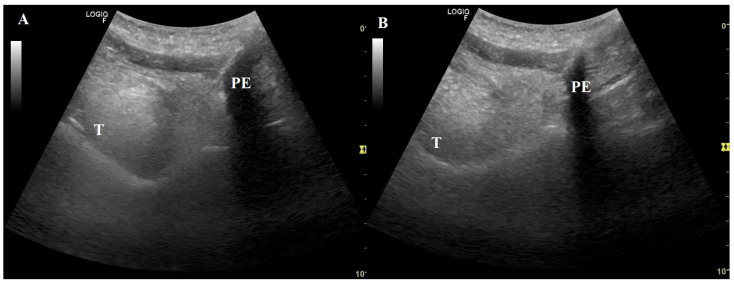
A patient affected by amyotrophic lateral sclerosis with no bulbar dysfunction; (**A**) background movement of tongue during insufflation. (**B**) Decreased diameter of pre-epiglottic space during exsufflation. Set parameters: insufflation pressure, +40 cmH_2_O; insufflation time, 1.5 s; high flow; exsufflation pressure, −40 cm_2_O; exsufflation time, 2.5 s; peak cough flow generated, 4.7 L/s. T = tongue; PE = pre-epiglottic space.

**Figure 2:**
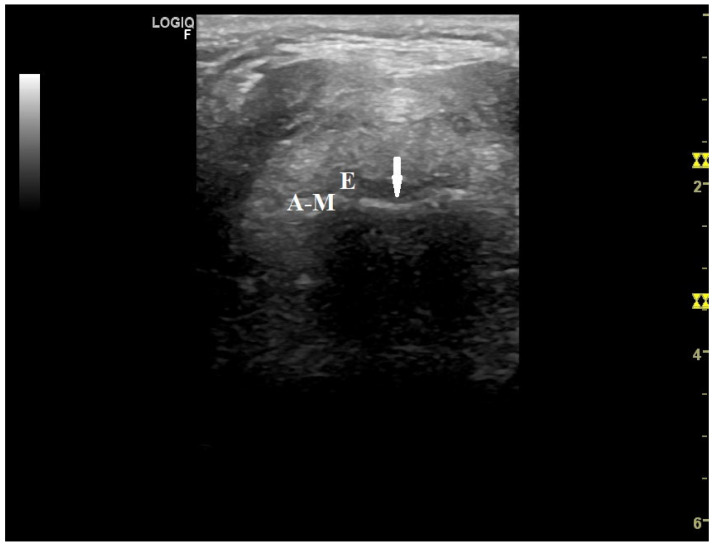
Background movement of epiglottis (arrow) during insufflation in a patient with amyotrophic lateral sclerosis and moderate bulbar dysfunction (Norris bulbar subscale 15). Set parameters: insufflation pressure, +45 cmH_2_O; insufflation time, 1.4 s; high flow; exsufflation pressure, −40 cm_2_O; exsufflation time, 2.5 s; peak cough flow generated, 4.2 L/s. E = epiglottis; A-M = air–mucosa interface.

**Figure 3:**
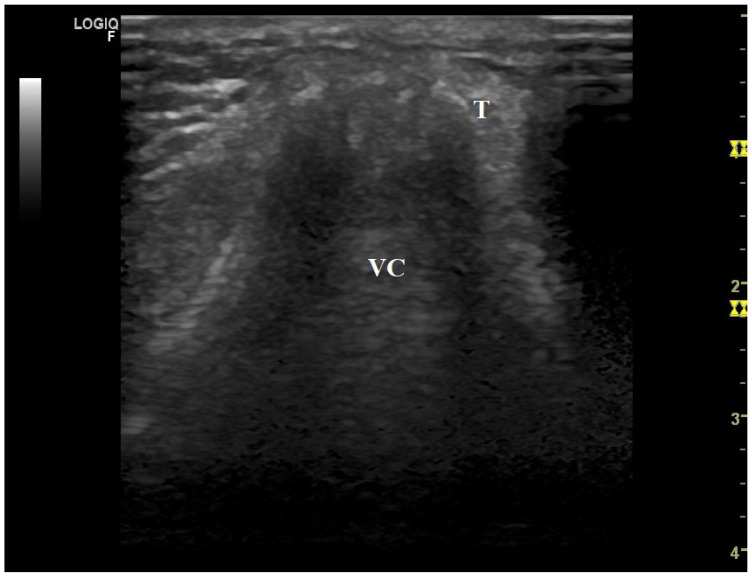
Glottis closure during insufflation in a patient with amyotrophic lateral sclerosis and severe bulbar dysfunction (Norris bulbar subscale 5). Set parameters: insufflation pressure, +40 cmH_2_O; insufflation time, 1.3 s; high flow; exsufflation pressure, −40 cm_2_O; exsufflation time, 3.0 s; peak cough flow generated, 2.6 L/s. VC = vocal cords; T = thyroid cartilage.

## Data Availability

All data and figures are included in this manuscript.
